# Tetracycline Resistant *Campylobacter jejuni* Subtypes Emanating from Beef Cattle Administered Non-Therapeutic Chlortetracycline are Longitudinally Transmitted within the Production Continuum but are Not Detected in Ground Beef

**DOI:** 10.3390/microorganisms8010023

**Published:** 2019-12-21

**Authors:** G. Douglas Inglis, Jenny F. Gusse, Kathaleen E. House, Tara G. Shelton, Eduardo N. Taboada

**Affiliations:** 1 Lethbridge Research and Development Centre, Agriculture and Agri-Food Canada, 5403-1st Avenue South, Lethbridge, AB T1J 4B1, Canada; Jenny.Gusse@canada.ca (J.F.G.); Kathaleen.House@canada.ca (K.E.H.); Tara.Shelton@canada.ca (T.G.S.); 2 National Microbiology Laboratory, Public Health Agency of Canada, 1015 Arlington Street, Winnipeg, MB R3E 3M4, Canada; Eduardo.Taboada@canada.ca

**Keywords:** beef cattle, *Campylobacter jejuni*, antimicrobial resistance, longitudinal transmission, clinically relevant subtypes, health risk

## Abstract

The impacts of the antimicrobial growth promoter (AGP), chlortetracycline with sulfamethazine (AS700), on the development of antimicrobial resistance and longitudinal transmission of *Campylobacter jejuni* within the beef production continuum were empirically determined. Carriage of tetracycline resistance determinants in the enteric bacterial community increased at a greater rate for AS700-treatment cattle. The majority of the bacteria from animals administered AS700 carried *tetW*. Densities of *C. jejuni* shed in feces increased over the confined feeding period, and the administration of AS700 did not conspicuously reduce *C. jejuni* densities in feces or within the intestine. The majority of *C. jejuni* isolates recovered were resistant to tetracycline, but the resistance rates to other antibiotics was low (≤20.1%). The richness of *C. jejuni* subtypes recovered from AS700-treated animals that were either resistant or susceptible to tetracycline was reduced, indicating selection pressure due to AGP administration. Moreover, a degree of subtype-specific resistance to tetracycline was observed. *tetO* was the primary tetracycline resistance determinant conferring resistance in *C. jejuni* isolates recovered from cattle and people. Clinically-relevant *C. jejuni* subtypes (subtypes that represent a risk to human health) that were resistant to tetracycline were isolated from cattle feces, digesta, hides, the abattoir environment, and carcasses, but not from ground beef. Thus, study findings indicate that clinically-relevant *C. jejuni* subtypes associated with beef cattle, including those resistant to antibiotics, do not represent a significant foodborne risk.

## 1. Introduction

Campylobacteriosis is the a common foodborne bacteria disease in Canada and elsewhere, primarily the result of infection by *Campylobacter jejuni* [1]. Southwestern Alberta (SWA) is a region with a high rate of campylobacteriosis. Rates of campylobacteriosis caused by *C. jejuni* (59.7 cases/100 K) in this region are substantially higher than the provincial (28.5 cases/100 K) and national (30.0 cases/100 K) averages [2,3,4]. A salient feature of SWA is the high density of livestock, and in particular, beef cattle. In this regard, there are ≈1200 K beef cattle with 51% in confined feeding operations (CFOs) in the region [5]. There were ≈181 K people living in SWA in 2016 with a ≈40:60 rural:urban distribution. Reasons for the high rates of campylobacteriosis in the region are currently uncertain. However, a clinical study of campylobacteriosis conducted over a 1-year period indicated that temporal clusters contributed to cases of campylobacteriosis, and a majority of infections by *C. jejuni* (70.3%) were linked to subtypes associated with beef cattle [6]. *Campylobacter jejuni* readily colonizes the intestinal tract of cattle, and is regularly shed in large numbers in feces [7,8,9,10,11,12]. Some evidence suggests that direct contact with cattle (i.e. occupational risk) contributes to the burden of campylobacteriosis [13]. The degree to which the consumption of beef contaminated with viable *C. jejuni* contributes to disease in SWA and elsewhere is currently unknown. A number of studies have conducted snapshot examinations of *C. jejuni* associated with carcasses [14,15,16,17] and retail beef [18,19,20,21], but few have longitudinally examined the transmission of *C. jejuni* subtypes throughout the beef production continuum to date, or examined the risk posed to people. Our research and that of others has shown that only a subset of *C. jejuni* subtypes are associated with human infections. This emphasizes the necessity of examining the epidemiology of the bacterium at a subtype level of resolution to ascertain the human health risk.

In North America, the majority of beef cattle are finished in confined feeding operations (CFOs), and until recently, cattle were frequently administered antimicrobial agents in feeds as antimicrobial growth promoters (AGPs). AGPs are administered at relatively low concentrations in feed for prolonged periods to promote growth [22]. Although the mechanisms of action of AGPs are poorly understood at present [23], selection for tetracycline resistance in *C. jejuni* occurs rapidly in beef cattle that are administered AGPs within CFOs [24,25]. Moreover, resistance to tetracycline has been shown to be subtype specific, consistent with the horizontal transmission of plasmids carrying resistance determinants under selection [25]. Of concern, we observed that resistance to fluoroquinolones increased in *C. jejuni* from beef cattle as a function of time within the CFO, resistance appeared to arise independent of selection pressure from antibiotic administration, and resistance was not subtype specific [25]. It is noteworthy that the World Health Organization recently listed fluoroquinolone-resistant *C. jejuni* as a salient risk to human health and the development of new antibiotics as a high priority [26]. Consistent with this conclusion, we have observed the rate of resistance to fluoroquinolones in clinical *C. jejuni* in SWA is increasing significantly in recent years. What impact AGP administration has on selection for and transmission of resistant *C. jejuni* subtypes associated with beef cattle is poorly understood. Furthermore, limited research to date has examined the risk to people by clinically-relevant subtypes (CRS) of the *C. jejuni* associated with beef cattle, including subtypes resistant to antibiotics. 

We hypothesized that: (i) resistance determinants are endemic at a low frequency within the enteric bacterial community of beef cattle; (ii) AGP administration will rapidly select for carriage of tetracycline and sulfamethazine resistant determinants within the enteric community; (iii) *C. jejuni* densities shed in beef cattle feces will not be affected by AGP treatment; (iv) *C. jejuni* subtypes, including those resistant to tetracycline will be longitudinally transmitted to carcasses and ground beef; (v) resistance to tetracycline will be subtype specific; and (vi) a subset of the *C. jejuni* subtypes associated with cattle will be transmitted to ground beef and pose an infection risk to human beings. To test these hypotheses, a replicated study using an experimental CFO in which beef cattle were administered no antibiotics or the AGP, chlortetracycline and sulfamethazine, as the sole antibiotics was conducted. Feces were temporally collected from cattle throughout the CFO period, and *C. jejuni* shed in feces and the carriage of resistance determinants within the enteric bacterial community were quantified. Cattle were longitudinally sampled throughout the slaughter process, *C. jejuni* associated with samples including ground beef generated from carcasses were quantified, and recovered *C. jejuni* isolates were genotyped and levels of resistance to antibiotics were measured to ascertain the transmission dynamics of subtypes, including those resistant to antibiotics from farm-to-fork. Moreover, *C. jejuni* isolates obtained from diarrheic people in SWA as a model agroecosystem were genotyped to ascertain the human health risk represented by *C. jejuni* subtypes associated with beef cattle.

## 2. Materials and Methods

### 2.1. Ethics Approvals

Before commencement of the study, the experiment was approved by the Lethbridge Research Centre Animal Care Committee, and all cattle involved in this study were cared for according to the guidelines set out by the Canadian Council on Animal Care [27]. Approval to transfer *Campylobacter* isolates from the Chinook Regional Hospital to the Agriculture and Agri-Food Canada Lethbridge Research and Development Centre was obtained from the University of Lethbridge Office of Research Ethics (Certificate of Human Subject Research #715; 01 September 2007) and the University of Alberta Research Ethics Office (Health Research Ethics Board #Pro00094238; 13 September 2019). Information that was transferred with the isolates was restricted to the isolate identifier, the year of isolation, and whether the bacterium was isolated from a stool or blood. No information that could reveal the identity of the infected individual was disclosed to research personnel.

### 2.2. Confined Feeding Operation

A detailed description of the CFO and sample collection is provided in Reti et al. [28]. Briefly, Angus-cross beef cattle were housed in an experimental CFO located at the Lethbridge Research and Development Centre. Calves arriving at the CFO were not previously exposed to antibiotics, and were assigned to one of two treatments: (1) no antimicrobial agents (i.e., control); and (2) 350 mg/head/d chlortetracycline and 350 mg/head/d sulfamethazine (AS700; Aureo S7 700 G, Alpharma Inc., Bridgewater Township, NJ, USA). The experiment was conducted as a randomized complete block design, with five replicates (i.e., blocks) per treatment. Each block consisted of a separate pen containing ten steers. During the CFO period, animals were fed a forage-based diet for the first 12 weeks (‘backgrounding’ period), transitioned to a grain-based diet (85% barley, 10% barley silage, 5% supplement) over a 3-week period, and then maintained on the grain-based diet for an additional 18 weeks (i.e., ‘finishing’ period). Cattle were fed once daily ensuring that all of the feed allotted to each pen was consumed. AS700 was introduced into the diets 5 days after the cattle arrived at the CFO, they were included in the diet for 27.5 weeks, and then removed from the diet 4 weeks prior to slaughter to meet the requisite withdrawal period. Besides AS700, none of the study cattle were administered other antimicrobial agents.

### 2.3. Sample Collection

Feces were collected from animals at 4-week intervals throughout the CFO period. Fecal samples were obtained per rectum as described previously [29]. The final fecal samples were obtained upon transport to the abattoir. After collection, fecal samples were placed on ice and processed within 4 h.

Fifteen steers from the control and AS700 treatments were selected for detailed sampling in the abattoir. The abattoir used was a provincial inspected medium capacity plant (Ben’s Quality Meats, Picture Butte, AB, Canada). Three animals were processed from each of the five replicate pens per treatment. Control animals were transported in the late afternoon of day one and euthanized on the morning of day two. AS700 animals were transported on the afternoon of day two and euthanized on the morning of day three. Following transport of the control animals, the stock trailer was thoroughly cleaned with a pressure washer. Animals were humanely euthanized according to industry standards, and within 5 min of euthanization, samples from the surface of the hide of all animals (brisket and rump) were sampled with a sterile 2- by 4-cm cellulose acetate sponge (Nasco Canada, Newmarket, ON, Canada) moistened with Columbia broth (CB; Difco, BD Biosciences, Mississauga, ON, Canada). The area sampled (i.e., 30 × 30 cm) was delimited using a sanitized wire frame. Air samples were obtained at a height of 1 m adjacent to the carcass during the hide removal process for each animal. To collect air samples, an inertial air sampler (MAS-100; EMD Chemicals Inc., Gibbstown, NJ, USA) containing a Petri dish filled with Karmali Agar (KA; Oxoid Inc., Nepean, ON, Canada) with selective supplement, SR167 (KKA; Oxoid Inc.) was operated for 2 min at 100 L/min. Following hide removal, evisceration, and breaking (i.e., the carcass was divided into two halves by cutting down the spinal column), the carcass was sprayed with warm water (38 to 43 °C) according to the standard operating procedures of the plant. After the carcass wash, the surface of one side of each carcass was sponge sampled in the same manner as the hides. Carcasses were then transferred to a chiller room (6 °C), taking care to ensure that they did not contact other carcasses. The surface of the other side of each carcass was sampled in the same manner after 24 h in the carcass chiller. All sponges were maintained in sterile bags on ice until they were processed (ca. 2–4 h). Five environmental locations were also sponge sampled before animals were processed each day. Standard sanitation procedures for the abattoir at the end of the day were implemented.

The intestinal tract of five animals per antimicrobial treatment (one animal per pen) was removed ≈10 min after death. Intestinal segments were obtained from the descending portion of the duodenum (i.e., following the cranial flexure), the proximal jejunum, the central jejunum, the distal jejunum, the ileum (10 cm before the ileal-cecal junction), the free end of the cecum, the central flexure of the ascending colon, the descending colon (20 cm before the sigmoid colon), and the rectum [28]. Before excision of the intestinal sections, bilateral ligatures were applied adjacent to the excision site to minimize external contamination of the tissues with digesta. Samples were placed in individual bags on ice, transported to the laboratory, and processed within 4 h.

Meat trimmings (20% fat) from the brisket and rump of the right side of the carcass of each animal were obtained after 1 week in the chiller, and ground beef was generated from each sample was obtained using a Porkert #32 Bolt-Down Manual Meat Grinder (5-mm-diameter plate). The grinder was dismantled and sanitized between samples. Multiple washes with sterile water were conducted after exposure to the sanitizer to ensure its removal. A subsample of ground beef (150 g) was placed in a sterile filtered stomacher bag (Model 400 bags, Seward Laboratory Systems Inc., Bohemia, NY, USA) and maintained on ice until processed. The remaining ground beef sample was placed at 5 °C for 1 week, and a subsample similarly placed in a stomacher bag on ice. Samples were processed ≈2 h after generating ground beef.

### 2.4. Isolation of Campylobacter jejuni

Intestinal segments were aseptically excised and digesta was removed as described previously [30]. Feces and digesta were placed in a 10 times volume of CB and the sample homogenized. Mucosal surfaces were gently washed with chilled phosphate buffer to remove digesta, and sponges and mucosal segments were stomached in 20 mL of CB for 2 min on high setting using a Stomacher 80 (Seward Laboratory Systems Inc.). Sponges and mucosal segments were removed, the suspension centrifuged (14,900× *g* for 10 min), the supernatant removed, and the pellet re-suspended in 1 mL of CB. Ground beef was stomached in 200 mL of CB for 2 min on high setting using a Stomacher 400 (Seward Laboratory Systems Inc.), and the suspension was concentrated by centrifugation as for the sponge samples. An aliquot (25 µL) of the homogenate/stomached suspension was spread in duplicate onto *Campylobacter* blood-free selective medium (CCDA) with CCDA selective supplement CM739 (CS; Oxoid Inc.), and KKA, and maintained in a microaerobic atmosphere at 42 °C for 48 and 72 h as described previously [30]. Samples from ground beef were enriched in Bolton Broth (Oxoid Inc.) with selective supplement, SR0183 (BBS; Oxoid Inc.) [30]. Where applicable, cell biomass from each of two colonies per colony morphology characteristic of *Campylobacter* were streaked onto KA, and maintained at 37 °C in a microaerobic atmosphere. All presumptive *Campylobacter*-like isolates were streaked for purity, biomass suspended in CB containing 40% glycerol, and maintained frozen at −80 °C until identified.

### 2.5. Identification of Campylobacter jejuni

Genomic DNA of presumptive *Campylobacter* isolates was extracted using an AutoGen 740 robot (AutoGen, Inc., Holliston, MA, USA) according to the manufacturer’s protocol for Gram negative bacteria. Isolates were identified by PCR using *Campylobacter* genus-specific [31] and *C. jejuni*-specific [32] PCR.

### 2.6. Susceptibility to Antimicrobials

The susceptibility of *C. jejuni* isolates to seven antimicrobial agents was quantified. The minimum inhibitory concentrations (MIC) to ciprofloxacin, chloramphenicol, clindamycin, erythromycin, gentamicin, nalidixic acid, and tetracycline were determined using the agar dilution methodology according to Clinical and Laboratory Standards Institute (CLSI) standards [33] with the exception that the Mueller–Hinton agar (Difco, Sparks, Maryland, USA) was not amended with 5% defibrinated horse blood. *Campylobacter* cells were harvested from the surface of the medium after 48 h growth under microaerobic conditions at 37 °C. Cells were suspended in sterile NaCl (0.075%), and the density of cells was adjusted to a 0.5 McFarland standard using a spectrophotometer (Genesys 20, Thermo Scientific, Rockford, IL, USA; 625 nm). Aliquots (300 µL) of the saline suspension were pipetted into the seeding wells of a Cathra replicator (Oxoid Inc.). Freshly prepared plates of Mueller–Hinton agar amended with antimicrobial agents were then inoculated using 1-mm pins in the inoculating head of the replicator. Cultures were incubated in the microaerobic atmosphere at 37 °C for 48 h, and the MIC was defined as the lowest concentration resulting in complete inhibition of visible growth on the medium. *Campylobacter jejuni* (ATCC 33560) was utilized as a quality control strain. The breakpoint MIC for resistance (intermediate resistance breakpoint in parentheses) was ≥4 µg/mL (2 µg/mL) for ciprofloxacin, ≥32 µg/mL (16 µg/mL) for chloramphenicol, ≥8 µg/mL (4 µg/mL) for clindamycin, ≥32 µg/mL (16 µg/mL) for erythromycin, ≥8 µg/mL (4 µg/mL) for gentamicin, ≥64 µg/mL (32 µg/mL) for nalidixic acid, and ≥16 µg/mL (8 µg/mL) for tetracycline [34].

### 2.7. Genotyping of Campylobacter jejuni and Human Health Risk Assessment

All recovered *C. jejuni* isolates were fingerprinted using a 40-locus comparative genomic fingerprinting (CGF) method [30,35]. To ascertain health risk of *C. jejuni* subtypes, the CGF40 profiles of tetracycline resistant and susceptible subtypes recovered from beef cattle and diarrheic human beings in the study region from 2004 to 2017 (*n* = 1171) were compared; the susceptibility of isolates recovered from people to tetracycline was determined using the same agar dilution method protocol with a breakpoint MIC of ≥16 μg/mL [25,36]. In addition, CGF40 profiles of prominent tetracycline resistant and susceptible subtypes associated with both cattle and human beings were queried against the profiles of subtypes within the Canadian *Campylobacter* comparative genomic fingerprinting database (C^3^GFdb), which contains >25,000 fingerprinted *C. jejuni* isolates from human beings, livestock, wildlife, and environmental matrices in Alberta and elsewhere in Canada.

### 2.8. Storage and DNA Extraction from Samples

Aliquots of feces and digesta (200 ± 5 mg) and mucosal biopsies (5-mm diameter) were stored frozen at −20 °C. For sponge samples concentrated by centrifugation, 200 µL aliquots were frozen at −20 °C. Aliquots (200 µL) from carcasses and ground beef were also treated with ethidium monoazide before freezer storage as described previously [37]. Genomic DNA was extracted from feces, digesta, and sponge washes using a QIAamp DNA stool minikit (Qiagen Inc., Toronto, ON, Canada) according to the manufacturer’s specifications for pathogen detection. For mucosal tissues, the QIAGEN DNeasy tissue kit (Qiagen Inc.) was used according to the manufacturer’s recommendation. An internal amplification control (IAC) was added to all substrates before they were extracted [8].

### 2.9. Quantification of Campylobacter jejuni

*Campylobacter jejuni* in/on feces, digesta, carcasses, and ground beef was quantified by PCR as described previously [30,38]. Briefly, a Stratagene Mx3005 (Stratagene Products, La Jolla, CA, USA) was used. All reactions were run in duplicate, and the mean value of the observations was used for analysis. Standard curves were established using genomic DNA from known concentrations of *C. jejuni* NCTC 11168. The number of *C. jejuni* cells was expressed as log_10_ copy number/g of feces or digesta, and log_10_ copy number/cm^2^ of mucosal, hides, or carcasses. For all reactions, melt curve analysis was conducted to confirm amplification specificity.

### 2.10. Quantification of Total Bacteria

Quantification of total bacteria was conducted as described previously [39]. Briefly, extracted DNA was amplified using QuantiTect SYBR Green (Qiagen Inc.) and the primers, HDA1 and HDA2; these primers target the 16S rRNA gene. The reaction mix (1X) consisted of 10 µL 2X QuantiTect SYBR Green Mastermix, 1.8 µL HDA1 (10 µM), 1.2 µL HDA2 (10 µM), 0.1 μg/μL BSA, 3 µL nuclease free water (Qiagen Inc.) and 2 µL of template. Samples were amplified and fluorescence detected on a Mx3005p thermocycler (Agilent, Missassauga, ON, Canada). Thermocycler conditions consisted of an activation cycle of 95 °C for 15 min, followed by 40 cycles at 94 °C for 15 s, 56 °C for 30 s, and 72 °C for 30 s. Optimization of quantitative PCR (qPCR) reaction conditions was completed using *Escherichia coli* (ATCC 25922) in a five times dilution series, with 20 ng per reaction being the highest DNA concentration. This isolate was also used as a quantitative standard for each qPCR run, with 1 ng of *E. coli* DNA being equal to 1.4 × 10^6^ genome copies. All reactions were run in duplicate, and the mean value of the observations was used for analysis. Standard curves were established using genomic DNA from known concentrations of *E. coli* ATCC 25922, assuming three copies of the 16S rRNA gene per cell. The number of bacteria was expressed as log_10_ copy number/g of feces or digesta, and log_10_ copy number/cm^2^ of mucosa. For all reactions, melt curve analysis was conducted to confirm amplification specificity.

### 2.11. Quantification of Antimicrobial Resistance Determinants

Extracted DNA was subjected to qPCR for each antibiotic resistance determinant using QuantiTect SYBR Green (Qiagen Inc.) and the corresponding primer sets for *tetB* [40], *tetC* [41], *tetW* [41], *tetL* [40], *tetM* [41], *tetO* [41], and *sul2* [42]. Thermocycler conditions consisted of an activation cycle of 95 °C for 15 min, and 40 cycles at 94 °C for 15 s, the T_A_ for the respective primers for 30 s, and 72 °C for 30 s. For each qPCR run, DNA from a plasmid containing the determinant of interest was used as a positive control. Plasmids were constructed by amplifying the gene from a positive sample found in the study, ligating this product into a pDRIVE Cloning Vector (Qiagen Inc.) and transforming it into QIAGEN EZ Competent Cells (Qiagen Inc.) according to manufacturers’ protocols. Plasmids were sequence verified for the insert. The number of determinants was expressed as log_10_ copy number/g of feces or digesta, and log_10_ copy number/cm^2^ of mucosal, hides, or carcasses. Carriage of determinants was also calculated relative to total bacterial densities (%).

### 2.12. Analyses

The mixed procedure of the Statistical Analyses Software (SAS; Cary, NC, USA) was used. To analyze the temporal (e.g., fecal collection time) and spatial (e.g., intestinal location) effects, repeated measures was used, and the appropriate covariance structure was utilized according to the lowest Akaike’s information criterion. The least squares means test was used to evaluate differences among means of interest. Isolates were assigned to CGF subtype clusters at 100% and 95% levels of resolution using the simple matching analysis coefficient with unweighted pair group method with arithmetic mean (UPGMA) clustering in Bionumerics (version 6.6, Applied Maths, Austin, TX, USA). Population structures were visualized as minimum spanning trees (MSTs) using Bionumerics (version 6.6, Applied Maths). Venn diagrams of subtypes were generated using pivot tables at a 95% level of resolution, including subtypes from cattle administered AS700 or no antibiotics, and those resistant and susceptible to tetracycline. The Fisher’s exact test was used to compare *C. jejuni* subtype counts between the AS700 and control treatments, and subtype counts that were resistant and susceptible to tetracycline.

## 3. Results

### 3.1. Time in the CFO, But Not Antibiotic Administration, Affected Densities of Bacteria Shed in Beef Cattle Feces 

**Figure 1:**
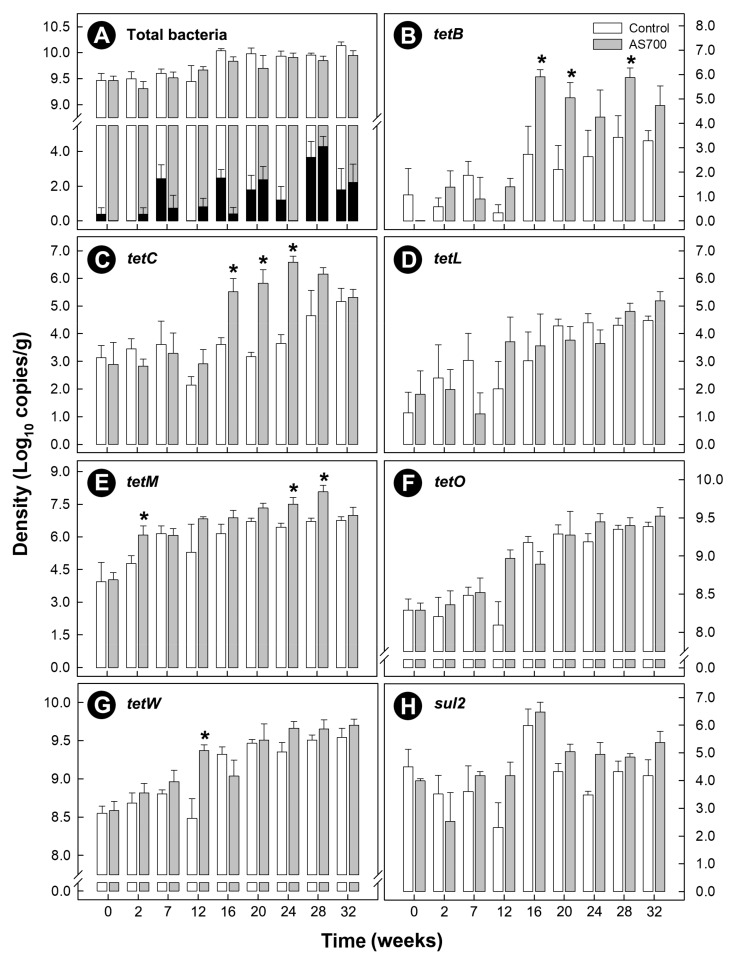
Densities of total bacteria and resistance determinants in feces from beef cattle administered chlortetracycline and sulfamethazine (AS700) or no antibiotics in feed, and housed in a beef cattle confined feeding operation for 32 weeks. After 28 weeks, administration of AS700 ceased for 4 weeks to meet mandatory withdrawal requirements. Densities of total bacteria (**A**), *tetB* (**B**), *tetC* (**C**), *tetL* (**D**), *tetM* (**E**), *tetO* (**F**), *tetW* (**G**), and *sul2* (**H**). Black histogram bars are *Campylobacter jejuni* densities. Vertical lines with histogram bars represent standard errors of the mean (*n* = 5). AS700 treatment histogram bars indicated with an asterisk (*) differ (*p* < 0.050) from the control treatment at the corresponding sample time.

The density of total bacteria shed in beef cattle feces increased over time (*p* < 0.001), and there was no difference (*p* = 0.124) between the control and AS700 treatments (Figure 1A). Cattle frequently shed *C. jejuni* in their feces throughout the CFO period, and the density of *C. jejuni* shed in feces increased over time (*p* < 0.001), but there was no difference between the control and AS700 treatments (*p* = 0.078) (Figure 1A).

### 3.2. Carriage of Antimicrobial Resistance Determinants was Common within the Fecal Bacterial Community

**Figure 2:**
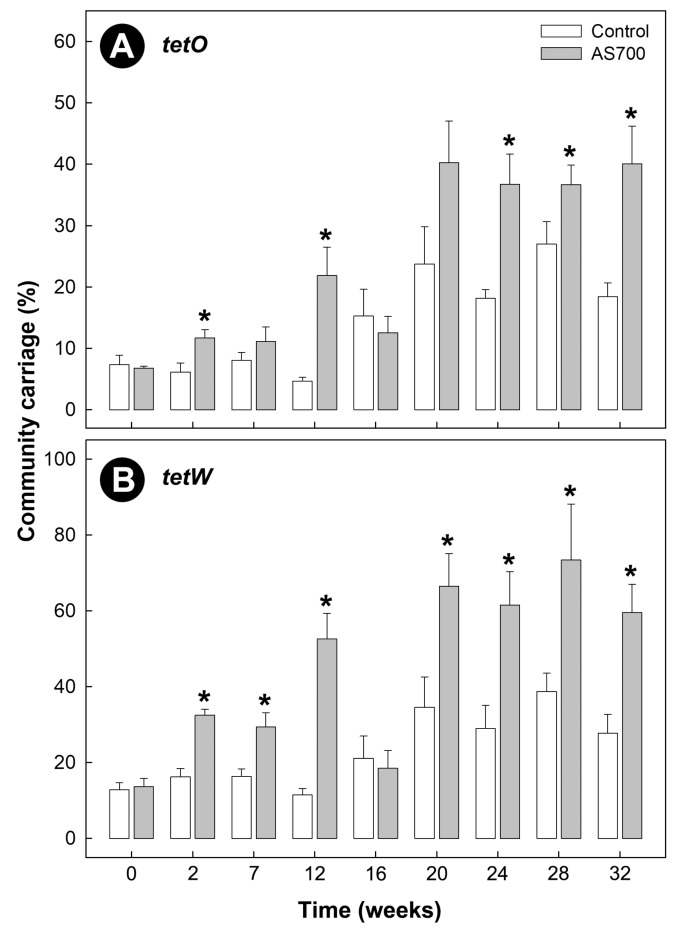
Community carriage of *tetO* (**A**) and *tetW* (**B**) in feces from beef cattle administered chlortetracycline and sulfamethazine (AS700) or no antibiotics in feed, and housed in a beef cattle confined feeding operation for 32 weeks (i.e., as a percentage of total bacteria). After 28 weeks, administration of AS700 ceased for 4 weeks to meet mandatory withdrawal requirements. Vertical lines with histogram bars represent standard errors of the mean (*n* = 5). AS700 treatment histogram bars indicated with an asterisk (*) differ (*p* < 0.050) from the control treatment at the corresponding sample time.

Carriage of *tetB*, *tetC*, *tetL*, *tetM*, *tetO*, and *tetW* was observed within the fecal community of both Control and AS700 cattle, and increased over time (*p* < 0.001) regardless of treatment (Figure 1B–H). A higher carriage of *tetB* (*p* < 0.001), *tetC* (*p* < 0.001), *tetM* (*p* = 0.010), and *tetW* (*p* = 0.006) by bacteria in feces was observed in animals administered AS700 relative to control animals; however, the difference between the treatments for *tetW* were the result of a single time point (i.e., week 12). There was no effect of AS700 administration (*p* ≥ 0.084) on bacterial community carriage rates of *tetL* or *tetO* determinants. The two most abundant tetracycline resistance determinants observed were *tetO* and *tetW* (Figure 1F,G). AS700 administration increased (*p* ≤ 0.005) the percentage of the community that carried *tetO* and *tetW* over time relative to control animals (Figure 2A,B). Carriage of *tetO* and *tetW* determinants as a percentage of total bacteria from AS700 animals indicated that ≈40% and ≈75% of the community carried these determinants, respectively. Withdrawal of AS700 for 4 weeks before animal euthanization (i.e., between week 28 and 32) did not affect carriage of resistance determinants. In contrast, carriage of other *tet* determinants were observed in less than 5% of the community. Carriage of *sul2* was observed in the fecal bacterial community, but there was no difference in carriage rates (*p* = 0.059) between the AS700 and control treatments, and although there was a time effect (*p* < 0.001), there was not a conspicuous increase in carriage of *sul2* over the duration of the CFO period (Figure 1H).

### 3.3. Carriage of Resistance Determinants Was Common in Bacteria Throughout the Intestinal Tract

**Figure 3:**
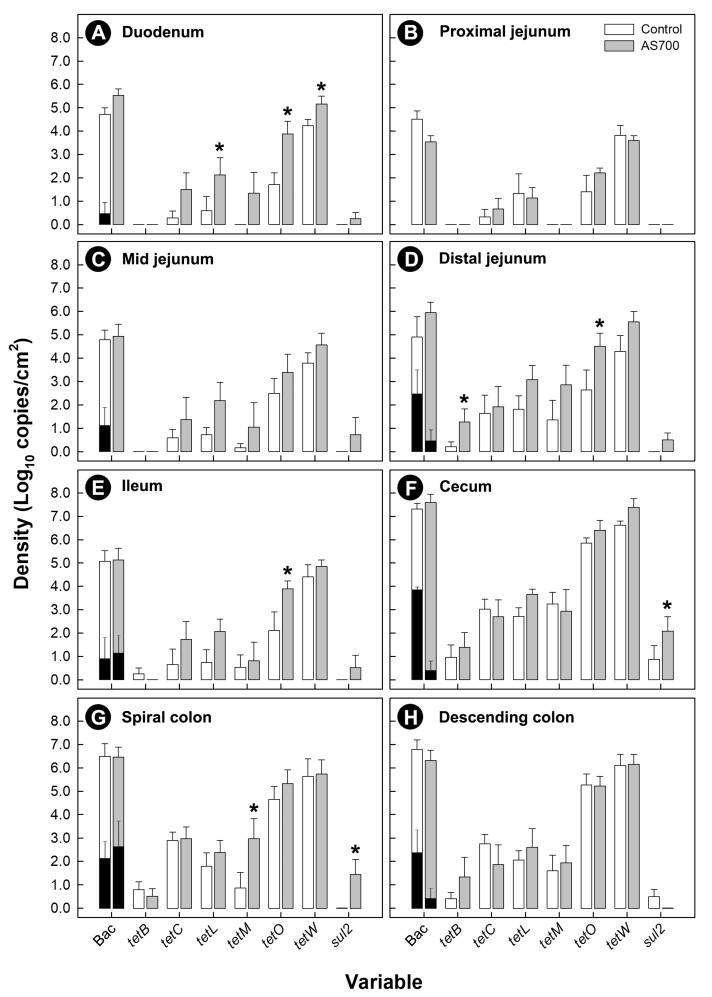
Densities of total bacteria (Bac) and resistance determinants (*tetB*, *tetC*, *tetL*, *tetM*, *tetO*, *tetW*, and *sul2*) associated with mucosa from the duodenum (**A**), proximal jejunum (**B**), mid-jejunum (**C**), distal jejunum (**D**), ileum (**E**), cecum (**F**), spiral colon (**G**), and descending colon (**H**) of beef cattle administered chlortetracycline and sulfamethazine (AS700) or no antibiotics in feed after euthanization. Black histogram bars are *Campylobacter jejuni* densities. Vertical lines with histogram bars represent standard errors of the mean (*n* = 5). AS700 treatment histogram bars indicated with an asterisk (*) differ (*p* < 0.050) from the control treatment at the corresponding variable.

Carriage of resistance determinants was common in bacteria associated with mucosa (Figure 3), and within digesta (Figure S1A–D). The carriage of various resistance determinants was higher (*p* ≤ 0.054) in bacteria from cattle treated with AS700, and particularly in bacteria associated with intestinal mucosa in the rectum (Figure S2). Although *C. jejuni* DNA was detected in digesta and associated with intestinal mucosa, it contributed less than 0.1% of the enteric community. At some intestinal locations, densities of *C. jejuni* cells were reduced (*p* ≤ 0.046) in animals administered AS700. The administration of AS700 did not affect densities (*p* = 0.475) of *C. jejuni* in digesta (Figure S1).

### 3.4. A High Prevalence of Campylobacter jejuni Isolates Were Resistant to Tetracycline

**Figure 4:**
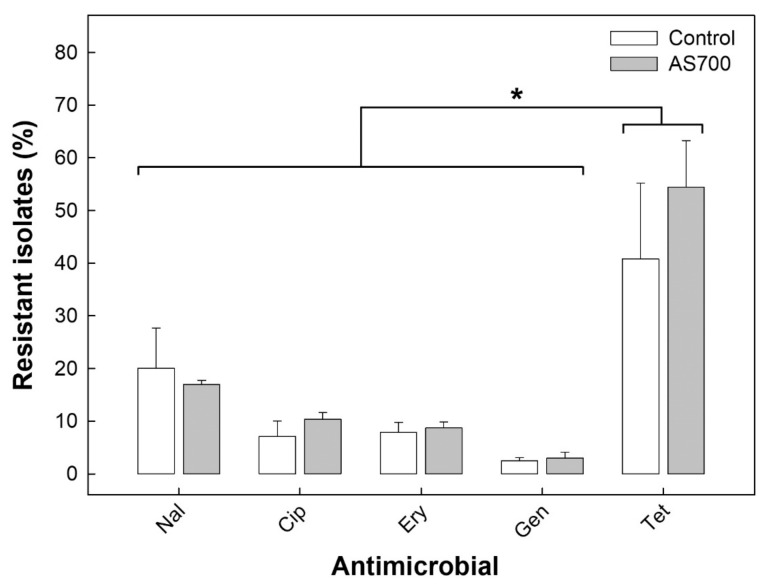
Prevalence of *Campylobacter jejuni* isolates resistant to nalidixic acid (Nal), ciprofloxacin (Cip), erythromycin (Ery), gentamicin (Gen), and tetracycline (Tet) recovered collectively from the abattoir environment, feces, intestine, hides, air, and carcasses from/of cattle administered chlortetracycline and sulfamethazine (AS700) or no antibiotics in the beef cattle confined feeding operation. Vertical lines with histogram bars represent standard errors of the mean (*n* = 5). There was no difference (*p* = 0.118) in rates of resistance to tetracycline between control and AS700 treatment animals. The asterisk (*) indicates that a higher (*p* ≤ 0.019) prevalence of *C. jejuni* isolates resistant to tetracycline relative to resistance to other antibiotics was observed regardless of AS700 administration.

Rates of resistance to tetracycline were substantially higher (*p* < 0.001) than resistance rates to nalidixic acid, ciprofloxacin, erythromycin, or gentamicin (Figure 4). All of the *C. jejuni* isolates examined carried the *tetO* resistance determinant, and not *tetB*, *tetC*, *tetL*, *tetM*, *tetQ*, or *tetW*. There was no effect (*p* = 0.998) of AS700 administration on the prevalence of *C. jejuni* isolates that were resistant to tetracycline. *Campylobacter jejuni* isolates resistant to tetracycline were recovered from the hides of cattle after euthanization and subsequently from the surfaces of carcasses after hide removal (Figure S3). There was no effect (*p* ≥ 0.429) of AS700 administration on resistance rates of *C. jejuni* isolates collectively or associated with individual samples. Evidence did not support transmission of resistant isolates between cattle housed in adjacent pens (Figure S4).

### 3.5. No Campylobacter jejuni Was Present in Ground Beef Generated from Contaminated Carcasses

No *C. jejuni* was isolated from ground beef generated from carcasses that were determined to be positive for the bacterium (Figure S3). Moreover, DNA from *C. jejuni* cells with an intact membrane was detected on carcasses (brisket and rump) immediately after euthanization and after storage at 4 °C for 24 h, but not in/on ground beef. Densities of *C. jejuni* DNA on carcasses ranged from 0.86 ± 0.26 to 2.02 ± 0.38 log_10_ copies/cm^2^.

### 3.6. Administration of AS700 to Cattle Reduced the Richness of Campylobacter jejuni Subtypes

**Figure 5:**
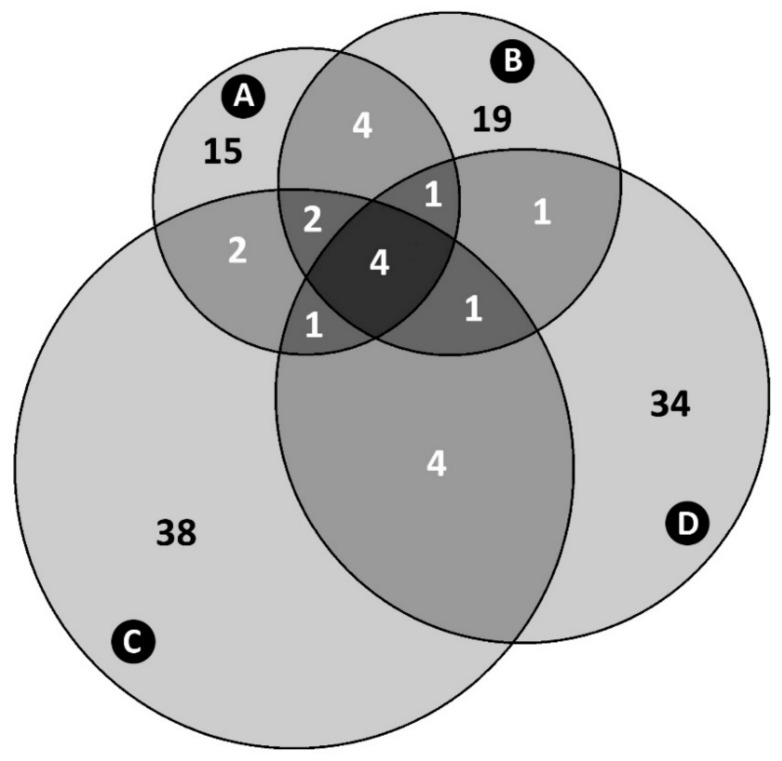
Four-way Venn diagram of *Campylobacter jejuni* comparative genomic fingerprinting (CGF) subtypes recovered from beef cattle throughout the production continuum (95% level of resolution). (**A**) Subtypes susceptible to tetracycline isolated from animals administered chlortetracycline and sulfamethazine (i.e., AS700 treatment). (**B**) Subtypes resistant to tetracycline isolated from AS700 treatment animals. (**C**) Subtypes susceptible to tetracycline isolated from animals not administered antibiotics (i.e., control treatment). (**D**) Subtypes resistant to tetracycline isolated from control treatment animals. Notably, the majority of subtypes contained *C. jejuni* isolates that were exclusively resistant or exclusively susceptible to tetracycline; subtypes that contained isolates that were both resistant and susceptible to tetracycline were less common. The total number of *C. jejuni* isolates examined and CGF subtypes obtained (95% level of resolution) are 664 and 126, respectively.

**Figure 6:**
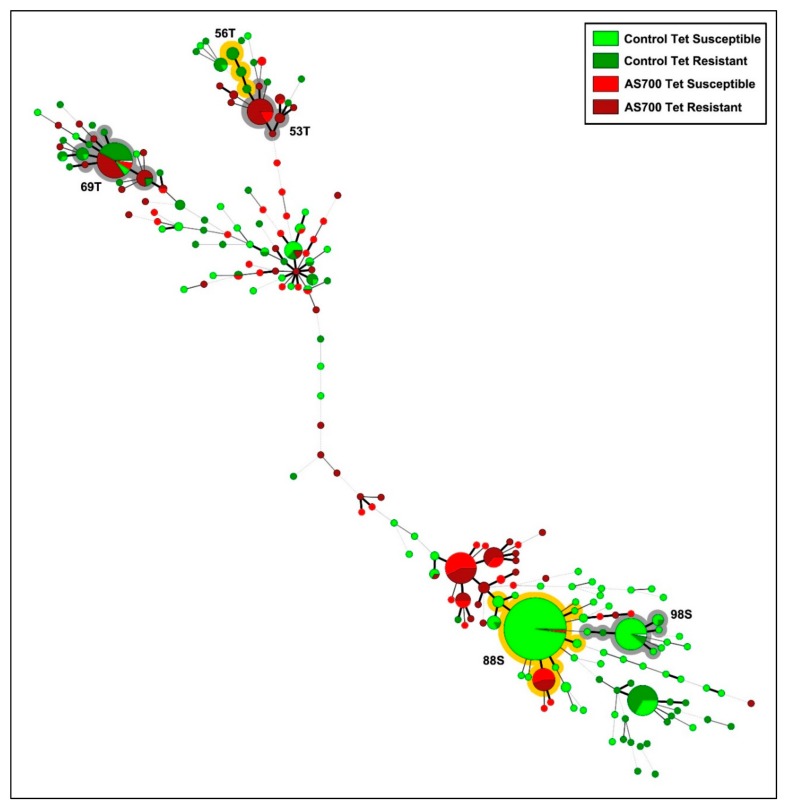
*Campylobacter jejuni* comparative genomic fingerprinting (CGF) subtypes recovered from beef cattle throughout the production continuum that were administered chlortetracycline and sulfamethazine (i.e., AS700 treatment) or no antibiotics (i.e., control treatment), and were resistant or susceptible to tetracycline. The minimum spanning tree was generated in Bionumerics (version 6.6, Applied Maths), and data were combined across pens and sample type. The size of the circle is proportional to the number of isolates within each CGF subtype (100% level of resolution), the thickness of lines connecting subtypes represent mismatched loci (i.e., one to three loci), and subtypes with no line represent ≥ four mismatched loci between respective subtypes. Shading illustrates subtype clusters that were predominantly tetracycline resistant (T) or tetracycline susceptible (S). Numbers associated with clusters indicate subtype cluster identifiers.

The number of *C. jejuni* subtypes recovered from animals administered AS700 that contained isolates that were resistant or susceptible to tetracycline was less (*p* < 0.001) than the number of resistant or susceptible subtypes from control animals (Figure 5). Some *C. jejuni* subtypes were differentially resistant or susceptible to tetracycline; for example, subtype clusters #53, #56, and #69 contained isolates that were predominately resistant to tetracycline, and clusters #88 and #98 contained isolates that were predominantly susceptible to tetracycline regardless of AS700 administration (Figure 6). Diverse subtypes resistant to ciprofloxacin were also observed, but no conspicuous selection of specific subtypes resistant to ciprofloxacin was detected (Figure S5). 

### 3.7. A Multitude of C. jejuni Subtypes Recovered from Beef Cattle Represent a High Health Risk to People But Not via consumption of Ground Beef

**Figure 7:**
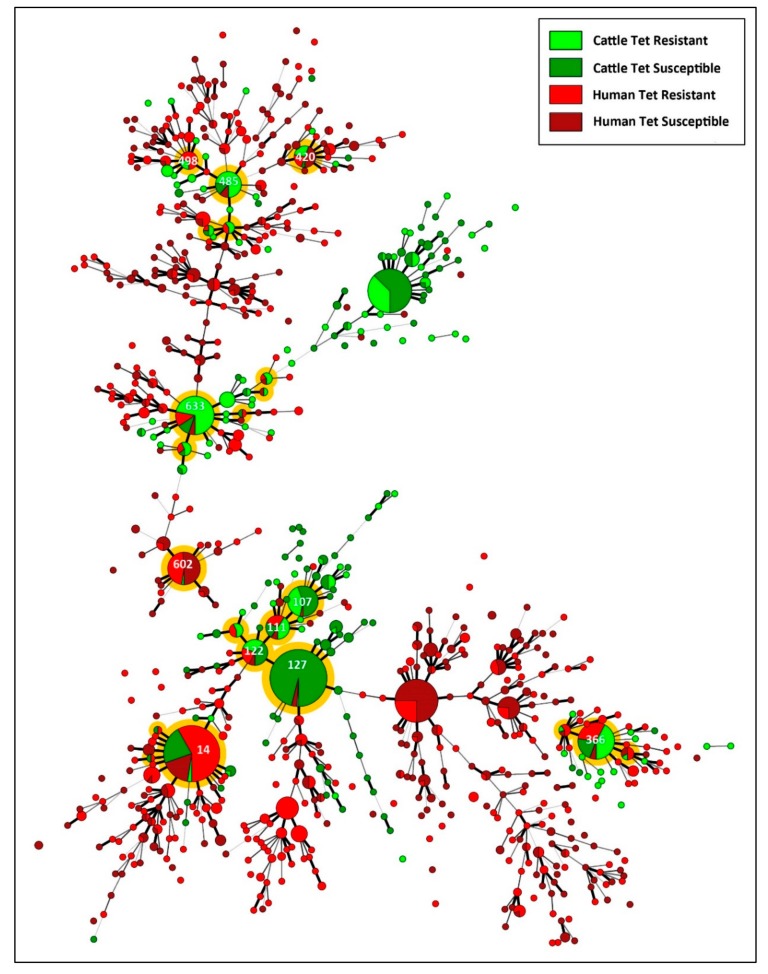
*Campylobacter jejuni* comparative genomic fingerprinting (CGF) subtypes from beef cattle in the current study that were resistant and susceptible to tetracycline, and from diarrheic human beings within the study area (2004 to 2017). The minimum spanning tree was generated in Bionumerics (version 6.6, Applied Maths). The size of the circle is proportional to the number of isolates within each CGF subtype (100% level of resolution), the thickness of lines connecting subtypes represent mismatched loci (i.e., one to three loci), and subtypes with no line represent ≥ four mismatched loci between respective subtypes. Shading illustrates subtype clusters that contained subtypes from both cattle and diarrheic people, and shaded clusters with numbers (i.e., subtype cluster identifiers) indicate clusters containing >10 isolates. The total number of isolates are 664 from cattle (285 tetracycline resistant and 379 tetracycline susceptible) and 1171 from diarrheic humans (631 tetracycline resistant and 540 tetracycline susceptible).

Of the *C. jejuni* isolates recovered from beef cattle and diarrheic humans in the study region, 22 CGF clusters contained isolates recovered from both hosts (i.e., CRS) (Figure 7). Of these, eleven contained more than ten isolates. Only one cluster (i.e., subtype cluster #14) was an identical match to isolates within the C^3^GFdb (subtype 0169.001.002); this subtype represents a large cluster within the C^3^GFdb that contains isolates that are prominent in Alberta, and were prominently recovered from human beings (30.8%), cattle (47.6%), and chickens (13.9%) in Canada. The other ten clusters were novel to the C^3^GFdb, but were closely related to subtypes within the database that were predominately associated with cattle as well as human beings. Similarly to *C. jejuni* isolates recovered from cattle, a high prevalence of isolates recovered from diarrheic people in SWA were resistant to tetracycline (>50%). Although most large CGF subtype clusters contained isolates that were both resistant and susceptible to tetracycline in approximately equal proportions, isolates within cluster #127 from cattle and people (*n* = 126) were predominately susceptible to tetracycline (99.2%); this cluster is most similar to *C. jejuni* subtype 0.238.002.008 and 0238.014.002 (95% similarity) within the C^3^GFdb.

## 4. Discussion

We hypothesized that resistance determinants are endemic at a low frequency within the enteric bacterial community of beef cattle, and AGP administration will rapidly select for carriage of tetracycline and sulfamethazine resistant determinants within the enteric community. Consistent with our hypothesis, we observed that the carriage of tetracycline resistant determinants (*tetB*, *tetC*, *tetM*, and *tetW*) increased over time within the fecal bacterial community for cattle administered AS700. Moreover, increased carriage of these tetracycline determinants, as well as *sul2*, were observed in bacteria associated with intestinal mucus and within digesta. Our findings indicate the AGP administration exerted selection pressure. This has been observed previously in feces of beef cattle and pigs administered AGPs [43,44]. Two proposed hypotheses of AGP action are the “microbiota modulation hypothesis” and the “immunomodulation hypothesis” [23]. The former hypothesizes that AGPs regulate bacteria within the intestinal community, which subsequently confers a health benefit to the host (e.g., competitive exclusion of pathogens). The latter hypothesizes that AGPs function by reducing immune responses within the intestine that are metabolically costly to the host, thereby providing a catabolic advantage to the host. As we observed, selection for carriage of antimicrobial resistance determinants within the intestinal bacterial community supports the microbiota modulation hypothesis. However, we also observed that the majority of the community carried *tetO* or *tetW*, which would be expected to confer phenotypic resistance in enteric bacteria [45,46]. Resistance to tetracycline is mediated through several mechanisms, which include tetracycline efflux, protection from binding of tetracycline to the ribosome via binding of specific cyptoplasmic proteins, or modification of 16S rRNA at the tetracycline-binding site [47]. In this regard, *tetO* and *tetW* confer resistance by interfering with the binding of aminoacyl-tRNA to the ribosomal site [48,49]. Both *tetO* and *tetW* resistance determinants are widespread within enteric bacteria of various mammals including ruminants [46,50]. Furthermore, these determinants are typically carried on mobile elements (e.g., plasmids) that facilitate horizontal transfer amongst bacteria of different phylogenies [51], followed by clonal expansion. That the majority of the enteric community of beef cattle were observed to carry *tetO* and *tetW* determinants in the current study is congruent with the immunomodulation hypothesis of AGP action (i.e., given the majority of bacteria are resistant, tetracycline administration would not be expected to provide a competitive advantage or disadvantage to specific bacterial taxa). Moreover, this observation supports empirical findings that AGPs directly or indirectly modulate the immune system [52]. It is very possible that the two hypothesized modes of AGP action act in concert, and elucidating the mechanisms of AGP action will facilitate the development of non-antibiotic alternatives that mimic their action. 

Before December 2018, tetracyclines were commonly administered to beef cattle in Canada as therapeutic and/or as metaphylactic (i.e., short-term mass treatment of a group of animals) agents, and as AGPs without a prescription from a veterinarian [25,53]. The administration of tetracyclines to beef cattle as a therapeutic and/or metaphylactic treatment is still permitted in Canada (i.e., with a prescription), but the administration of antibiotics in feed as an AGP is not. Moreover, AGPs are no longer permitted in beef cattle production in the USA, where cattle are primarily finished in CFOs in a similar manner to Canada. The administration of chlortetracycline as an AGP has previously been shown to select for resistance to tetracycline in *Campylobacter* species associated with beef cattle [29]. Thus, we hypothesized *C. jejuni* densities shed in beef cattle feces will not be reduced by AGP treatment. Although *C. jejuni* comprised a small percentage of the enteric microbiota (<0.001% of bacterial cells released in feces), we observed that the bacterium was consistently shed at relatively high densities in feces. This is consistent with previous studies that demonstrated that *Campylobacter* species, including *C. jejuni* are chronically shed in production cattle feces in large quantities [7,9,10,30]. Moreover, we observed that *C. jejuni* was consistently associated with mucosal surfaces throughout the intestinal tract of cattle regardless of AS700 treatment, and this corresponds with its microaerobic ecology [11]. Quantitative temporal shedding of *C. jejuni* cells in feces from cattle housed in CFO’s has not been previously studied to our knowledge. It is thought that the high prevalence of animals shedding *C. jejuni* is influenced by the close proximity of animals to each other, which facilitates horizontal transmission of the bacterium [25]. Given that individual cattle are colonized by diverse subtypes [30] coupled with high rates of horizontal transmission, it is reasonable to conclude that this translates to increased rates of shedding *C. jejuni* in cattle feces as a function of time that animals are in the CFO.

It has been proposed that a subset of calves entering CFO’s carry *C. jejuni* that are resistant to tetracycline, and under selection pressure within the CFO, the resistant subtypes differentially proliferate [25]. Moreover, individual beef cattle carry multiple subtypes of *C. jejuni* [30]. In the current study, we restricted our phenotypic resistance determinations of *C. jejuni* associated with cattle in the CFO to feces obtained from cattle just before euthanization, and from the intestine of animals immediately afterwards. We observed high rates of resistance in *C. jejuni* recovered from animals administered AS700, despite the 4-week AS700 withdrawal period preceding isolation. The removal of selection pressure for periods of time has similarly been observed not to affect resistance rates in *E. coli* recovered from beef cattle in a CFO [54]. Somewhat unexpectantly, the majority of *C. jejuni* isolates recovered from feces and the intestine of beef cattle in the current study were resistant to tetracycline regardless of AS700 administration (>50%). Furthermore, *C. jejuni* isolates longitudinally recovered from carcasses were equally resistant to tetracycline. In all of the *C. jejuni* isolates recovered from cattle that we examined, resistance was attributed to carriage of *tetO*, which is the most common determinant conferring tetracycline resistance in the bacterium associated with cattle in Canada [18,25]. One possibility is that resistant bacteria were transmitted from cattle administered AS700 to control animals maintained in pens in proximity to one another. However, an examination of *C. jejuni* subtypes recovered from individual animals by pen did not support this possibility. The high rates of resistance to tetracycline that we observed is consistent with the high rates of resistance in *C. jejuni* isolates recovered from diarrheic people in Alberta and elsewhere [55]. In this regard, rates of tetracycline resistant isolates found in people living in SWA have remained stable since we commenced monitoring in 2004, ranging from 41.6 to 65.1%. The degree to which tetracycline resistant *C. jejuni* isolates originating from cattle subsequently infect human beings in SWA is currently unknown. As indicated previously, the practice of AGP administration to beef cattle in Canada ended on December 1, 2018 [53], and it remains to be determined if this will reduce resistance rates in clinical strains of *C. jejuni*. However, administration of medically-important antimicrobials to beef cattle are still permitted in most jurisdictions under prescription from a licensed veterinarian, and different antimicrobial agents (e.g., tetracyclines, fluoroquinolones, macrolides) are often administered therapeutically to beef cattle in CFO’s [25].

Although a high prevalence of resistance to tetracycline was observed in the current study, rates of resistance to other antibiotics (i.e., a quinolone, fluoroquinolone, macrolide, and aminoglycoside) was conspicuously lower than to tetracycline. Given that only tetracycline and sulfamethazine were administered to cattle during their lifetime in the current study, the relatively low levels of resistance to other antibiotics, including to quinolones (17.0% resistance) and fluoroquinolones (8.7% resistance) was expected. The development of resistance to fluoroquinolones is a priority issue in human health [26], and we have observed that resistance to ciprofloxacin in *C. jejuni* infecting diarrheic people in SWA has increased substantially since 2004, peaking at ≈30% resistance in 2016 and beyond. The fluoroquinolone, enrofloxacin (i.e., Baytril^®^) is registered for therapeutic use in cattle in Canada against bovine respiratory disease, and the extent to which the therapeutic administration of enrofloxacin to cattle selects for resistant *C. jejuni* strains is currently unknown. The subcutaneous administration of enrofloxacin to mice colonized with *C. jejuni* for up to five days did not select for resistance in *C. jejuni* NCTC 11168 [32], suggesting that the fluoroquinolone resistance risk due to the therapeutic administration of enrofloxacin in cattle is low. However, Webb et al. [25] observed a significant increase in resistance to ciprofloxacin in *C. jejuni* subtypes from feedlot cattle over the period animals that were housed in commercial CFOs in southern Alberta that was not linked to antimicrobial agent administration. This raises questions as to non-antimicrobial agent factors that may select for resistance in *C. jejuni* subtypes. Further, Webb et al. [25] observed that fluoroquinolone resistance was subtype specific in *C. jejuni* from beef cattle in CFOs, which they attributed to the genetic mechanisms by which resistance is conferred. This is consistent with the limited diversity of subtypes resistant to fluoroquinolones observed in the current study. Additional research is required to understand the evolution of fluoroquinolone resistance in *C. jejuni* subtypes associated with cattle, and the risk that resistant subtypes represent to people. 

Primary goals of the current study were to ascertain the degree and mechanisms by which *C. jejuni* subtypes resistant to tetracycline are transmitted in the beef production continuum, and to determine the foodborne risk posed by the pathogen. We observed that tetracycline resistant and tetracycline susceptible *C. jejuni* subtypes associated with feces, intestinal digesta and mucosa, and hides were transmitted to carcasses during processing. Furthermore, the generation of aerosols containing *C. jejuni* during hide pulling contributed to the transmission of the bacterium to carcasses. High rates of resistance to tetracycline have been observed in *C. jejuni* isolates associated with beef carcasses [15,56] and meat in retail settings [57,58], but to our knowledge, limited research has addressed transmission. It is now recognized that not all subtypes of *C. jejuni* represent an equal risk of infection in people [6]. Therefore, we subtyped *C. jejuni* isolates in an attempt to shed light on resistance development, transmission mechanisms, and the risk posed to human beings. Although the overall prevalence of resistance to tetracycline was similar in *C. jejuni* originating from cattle administered AS700 and no antibiotics, AS700 greatly reduced the diversity of both resistant and susceptible subtypes of *C. jejuni*. Moreover, several prominent subtypes were differentially resistant or susceptible to tetracycline, and there was limited overlap between resistant and susceptible subtypes. This is consistent with an observation made by Webb et al. [25], who showed that *C. jejuni* subtypes resistant to tetracycline and shed in feces of cattle at low levels upon entrance of the animals to the CFO increased in prevalence within all animals during the CFO period, likely due to clonal expansion and horizontal transmission in the presence of selection pressure. Our findings emphasize that selection for resistance to tetracyclines and to other antibiotics in *C. jejuni*, and that the determinations of the risk represented need to be ascertained at a subtype level of resolution.

Southwestern Alberta is a region with high rates of campylobacteriosis [4,6]. In addition, the region has high densities of livestock, including >1 million head of cattle, with ≈50% of the cattle in CFOs [5]. In the current study, we observed that many of the *C. jejuni* subtypes recovered from cattle were also responsible for infections in people living in the study region. Although chickens are thought to be the primary reservoir of CRS [59], there are locations where this does not appear to be true. For example, in Finland, cattle appear to be an important primary reservoir of CRS [60]. Similar to the findings of the current study, a clinical study conducted in SWA over a 1-year period demonstrated that many of the *C. jejuni* subtypes infecting people in the region were primarily associated with cattle reservoirs [6]. Furthermore, the majority of infections were linked to spatial case clusters (i.e., “mini-outbreaks”). Although the consumption of untreated water and milk is a potential source of CRS of *C. jejuni* originating from cattle [61], the degree to which the consumption of beef contaminated with CRS contribute to the burden of campylobacteriosis has not been fully resolved at present. We detected viable *C. jejuni*, including CRS that were resistant to antibiotics on carcasses, albeit at relatively low densities (i.e., ≈10^3^ cells/cm^2^). However, we did not isolate any *C. jejuni* or detect *C. jejuni* DNA from ground beef generated from regions of carcasses (i.e., brisket and rump) that were deemed to be positive for the bacterium [30]. Considerable research has examined retail beef for *C. jejuni*, and they have concluded that viable *C. jejuni* cells are uncommon in retail beef in Canada and elsewhere [18,19,20,58,62,63,64,65,66]. This contrasts with the high prevalence of contamination of retail poultry by *C. jejuni* [67]. Our findings show that consumption of beef is not a significant risk for infection. This, coupled with evidence that CRS associated with cattle reservoirs are responsible for a high prevalence of human infections in SWA, indicate that non-foodborne mechanisms of transmission of *C. jejuni* subtypes originating from beef may contribute to infections in people. One possibility is occupational contact with cattle [13]. Another possibility is that cattle serve a crucial reservoir of CRS of *C. jejuni* that infect broiler chickens in production barns, which contaminate poultry meat during processing, and are subsequently consumed by and infect people.

## 5. Conclusions

We completed a longitudinal transmission study of *C. jejuni* throughout the beef cattle production continuum using SWA as a model agroecosystem. Cattle were exposed to the AGP, AS700, or no antibiotics, and the transmission of subtypes resistant to tetracycline was measured. Evidence for selection of resistant bacteria was observed within the enteric bacterial community, but high rates of resistance in *C. jejuni* were observed regardless of AGP administration. We showed that beef cattle were colonized by diverse *C. jejuni* subtypes, including a large number of subtypes that were resistant to tetracycline. Although the total number of *C. jejuni* cells shed in the intestine and shed in feces were equivalent in cattle administered AS700 relative to animals not administered antibiotics, the diversity of *C. jejuni* subtypes associated with AS700 animals was substantially lower. Hides, and subsequently, the surfaces of carcasses were contaminated with *C. jejuni*, but the bacterium was not detected in ground beef generated from contaminated carcasses. Although subtypes of *C. jejuni* recovered from beef cattle in the current study were associated with diarrheic people living in SWA, evidence from the study indicated that the foodborne risk from these subtypes in beef products, including those resistant to antimicrobial agents, is negligible. Thus, alternate mechanisms to foodborne transmission are likely responsible for the high rates of campylobacteriosis observed in SWA and elsewhere.

## Supplementary Materials

The following are available online at https://www.mdpi.com/2076-2607/8/1/23/s1.

## References

[1] Kaakoush N.O., Castano-Rodriguez N., Mitchell H.M., Man S.M. (2015). Global epidemiology of *Campylobacter* infection. Clin. Microbiol. Rev..

[2] Alberta Government Notifiable Diseases in Alberta 2004 Annual Report. Alta. Health Wellness..

[3] Public Health Agency of Canada Notifiable Diseases Online. http://diseases.canada.ca/notifiable/.

[4] Inglis G.D., Boras V.F., Houde A. (2011). Enteric campylobacteria and RNA viruses associated with healthy and diarrheic humans in the Chinook Health Region of southwestern Alberta, Canada. J. Clin. Microbiol..

[5] Alberta Government 2011 Census of Agriculture for Alberta. Alta. Agriclulture. Rural. Dev..

[6] Inglis G.D., Boras V.F., Webb A.L., Suttorp V.V., Hodgkinson P., Taboada E.N. (2019). Enhanced microbiological surveillance reveals that temporal case clusters contribute to the high rates of campylobacteriosis in a model agroecosystem. Int. J. Med. Microbiol..

[7] Inglis G.D., Kalischuk L.D. (2004). Direct quantification of *Campylobacter jejuni* and *Campylobacter lanienae* in feces of cattle by real-time quantitative PCR. Appl. Env. Microbiol..

[8] Inglis G.D., Kalischuk L.D. (2003). Use of PCR for direct detection of *Campylobacter* species in bovine feces. Appl. Env. Microbiol..

[9] Inglis G.D., Kalischuk L.D., Busz H.W. (2004). Chronic shedding of *Campylobacter* species in beef cattle. J. Appl. Microbiol..

[10] Inglis G.D., Kalischuk L.D., Busz H.W. (2003). A survey of *Campylobacter* species shed in faeces of beef cattle using polymerase chain reaction. Can. J. Microbiol..

[11] Inglis G.D., Kalischuk L.D., Busz H.W., Kastelic J.P. (2005). Colonization of cattle intestines by *Campylobacter jejuni* and *Campylobacter lanienae*. Appl. Environ. Microbiol..

[12] Thepault A., Poezevara T., Quesne S., Rose V., Chemaly M., Rivoal K. (2018). Prevalence of thermophilic *Campylobacter* in cattle production at slaughterhouse level in France and link between *C. jejuni* bovine strains and campylobacteriosis. Front. Microbiol..

[13] Hasselback P. (2002). Feedlot alley and enteric illness: Are they related or is southern Alberta just a wonderful place for humans, cattle and bugs to live?. Can. Lab. Med. Congr. Calg. ABCanada..

[14] Wysok B., Uradzinski J., Wojtacka J. (2015). Determination of the cytotoxic activity of *Campylobacter* strains isolated from bovine and swine carcasses in north-eastern Poland. Pol. J. Vet. Sci..

[15] Wieczorek K., Denis E., Lynch O., Osek J. (2013). Molecular characterization and antibiotic resistance profiling of *Campylobacter* isolated from cattle in Polish slaughterhouses. Food Microbiol..

[16] Van Donkersgoed J., Bohaychuk V., Besser T., Song X.M., Wagner B., Hancock D., Renter D., Dargatz D. (2009). Occurrence of foodborne bacteria in Alberta feedlots. Can. Vet. J..

[17] Hakkinen M., Heiska H., Hanninen M.L. (2007). Prevalence of *Campylobacter* spp. in cattle in Finland and antimicrobial susceptibilities of bovine *Campylobacter jejuni* strains. Appl. Env. Microbiol..

[18] Narvaez-Bravo C., Taboada E.N., Mutschall S.K., Aslam M. (2017). Epidemiology of antimicrobial resistant *Campylobacter* spp. isolated from retail meats in Canada. Int. J. Food. Microbiol..

[19] Llarena A.K., Sivonen K., Hanninen M.L. (2014). *Campylobacter jejuni* prevalence and hygienic quality of retail bovine ground meat in Finland. Lett. Appl. Microbiol..

[20] Zhao S., Young S.R., Tong E., Abbott J.W., Womack N., Friedman S.L., McDermott P.F. (2010). Antimicrobial resistance of *Campylobacter* isolates from retail meat in the United States between 2002 and 2007. Appl. Env. Microbiol..

[21] Hannon S.J., Inglis G.D., Allan B., Waldner C., Russell M.L., Potter A., Babiuk L.A., Townsend H.G.G. (2009). Prevalence and risk factor investigation of *Campylobacter* species in retail ground beef from Alberta, Canada. Food Prot. Trends.

[22] Cromwell G.L. (2002). Why and how antibiotics are used in swine production. Anim. Biotechnol..

[23] Brown K., Uwiera R.R., Kalmokoff M.L., Brooks S.P., Inglis G.D. (2017). Antimicrobial growth promoter use in livestock: A requirement to understand their modes of action to develop effective alternatives. Int. J. Antimicrob. Agents.

[24] Inglis G.D., Morck D.W., McAllister T.A., Entz T., Olson M.E., Yanke L.J., Read R.R. (2006). Temporal prevalence of antimicrobial resistance in *Campylobacter* spp. from beef cattle in Alberta feedlots. Appl. Environ. Microbiol..

[25] Webb A.L., Selinger L.B., Taboada E.N., Inglis G.D. (2018). Subtype-specific selection for resistance to fluoroquinolones but not to tetracyclines is evident in *Campylobacter jejuni* isolates from beef cattle in confined feeding operations in Southern Alberta, Canada. Appl. Environ. Microbiol..

[26] World Health Organization WHO Publishes List of Bacteria for which New Antibiotics are Urgently Needed. http://www.who.int/mediacentre/news/releases/2017/bacteria-antibiotics-needed/en/.

[27] Canadian Council on Animal Care Guidelines on: Laboratory Animal Facilities—Characteristics, Design and Development. https://www.ccac.ca/Documents/Standards/Guidelines/Facilities.pdf.

[28] Reti K.L., Thomas M.C., Yanke L.J., Selinger L.B., Inglis G.D. (2013). Effect of antimicrobial growth promoter administration on the intestinal microbiota of beef cattle. Gut Pathog..

[29] Inglis G.D., McAllister T.A., Busz H.W., Yanke L.J., Morck D.W., Olson M.E., Read R.R. (2005). Effects of subtherapeutic administration of antimicrobial agents to beef cattle on the prevalence of antimicrobial resistance in *Campylobacter jejuni* and *Campylobacter hyointestinalis*. Appl. Environ. Microbiol..

[30] Inglis G.D., Gusse J.G., House K.E., Shelton T.G., Taboada E.N. (2020). Clinically-relevant *Campylobacter jejuni* subtypes are readily found and transmitted within the cattle production continuum but present a limited foodborne risk. Appl. Environ. Microbiol..

[31] Linton D., Owen R.J., Stanley J. (1996). Rapid identification by PCR of the genus *Campylobacter* and of five *Campylobacter* species enteropathogenic for man and animals. Res. Microbiol..

[32] Inglis G.D., Zaytsoff S.J.M., Selinger L.B., Taboada E.N., Uwiera R.R.E. (2018). Therapeutic administration of enrofloxacin in mice does not select for fluoroquinolone resistance in *Campylobacter jejuni*. Can. J. Microbiol..

[33] Clinical and Laboratory Standards Institute (2006). Methods for antimicrobial dilution and disk susceptibility testing of infrequently isolated or fastidious bacteria; approved guideline. M45-A. Clin. Lab. Stand. Inst..

[34] National Animal Health Monitoring System Feedlot Report Part III: Health Management and Biosecurity in US Feedlots. http://www.aphis.usda.gov/animal_health/nahms/.

[35] Taboada E.N., Ross S.L., Mutschall S.K., Mackinnon J.M., Roberts M.J., Buchanan C.J., Kruczkiewicz P., Jokinen C.C., Thomas J.E., Nash J.H. (2012). Development and validation of a comparative genomic fingerprinting method for high-resolution genotyping of *Campylobacter jejuni*. J. Clin. Microbiol..

[36] National Antimicrobial Resistance Monitoring System (2010). NARMS 2010 Executive Report. https://www.fda.gov/downloads/animalveterinary/safetyhealth/antimicrobialresistance/nationalantimicrobialresistancemonitoringsystem/ucm312360.pdf.

[37] Inglis G.D., McAllister T.A., Larney F.J., Topp E. (2010). Prolonged survival of *Campylobacter* species in bovine manure compost. Appl. Env. Microbiol..

[38] Lone A.G., Selinger L.B., Uwiera R.R., Xu Y., Inglis G.D. (2013). *Campylobacter jejuni* colonization is associated with a dysbiosis in the cecal microbiota of mice in the absence of prominent inflammation. PLoS ONE.

[39] Abnous K., Brooks S.P., Kwan J., Matias F., Green-Johnson J., Selinger L.B., Thomas M., Kalmokoff M. (2009). Diets enriched in oat bran or wheat bran temporally and differentially alter the composition of the fecal community of rats. J. Nutr..

[40] Peak N., Knapp C.W., Yang R.K., Hanfelt M.M., Smith M.S., Aga D.S., Graham D.W. (2007). Abundance of six tetracycline resistance genes in wastewater lagoons at cattle feedlots with different antibiotic use strategies. Environ. Microbiol..

[41] Wu N., Qiao M., Zhang B., Cheng W.D., Zhu Y.G. (2010). Abundance and diversity of tetracycline resistance genes in soils adjacent to representative swine feedlots in China. Environ. Sci. Technol..

[42] Lanz R., Kuhnert P., Boerlin P. (2003). Antimicrobial resistance and resistance gene determinants in clinical *Escherichia coli* from different animal species in Switzerland. Vet. Microbiol..

[43] Kalmokoff M., Waddington L.M., Thomas M., Liang K.L., Ma C., Topp E., Dandurand U.D., Letellier A., Matias F., Brooks S.P. (2011). Continuous feeding of antimicrobial growth promoters to commercial swine during the growing/finishing phase does not modify faecal community erythromycin resistance or community structure. J. Appl. Microbiol..

[44] Alexander T.W., Yanke J.L., Reuter T., Topp E., Read R.R., Selinger B.L., McAllister T.A. (2011). Longitudinal characterization of antimicrobial resistance genes in feces shed from cattle fed different subtherapeutic antibiotics. BMC Microbiol.

[45] Aminov R.I., Garrigues-Jeanjean N., Mackie R.I. (2001). Molecular ecology of tetracycline resistance: Development and validation of primers for detection of tetracycline resistance genes encoding ribosomal protection proteins. Appl. Environ. Microbiol..

[46] Scott K.P., Melville C.M., Barbosa T.M., Flint H.J. (2000). Occurrence of the new tetracycline resistance gene *tet*(W) in bacteria from the human gut. Antimicrob. Agents. Chemother..

[47] Li W., Atkinson G.C., Thakor N.S., Allas U., Lu C.C., Chan K.Y., Tenson T., Schulten K., Wilson K.S., Hauryliuk V. (2013). Mechanism of tetracycline resistance by ribosomal protection protein Tet(O). Nat. Commun..

[48] Chopra I., Roberts M. (2001). Tetracycline antibiotics: Mode of action, applications, molecular biology, and epidemiology of bacterial resistance. Microbiol. Mol. Biol. Rev..

[49] Connell S.R., Tracz D.M., Nierhaus K.H., Taylor D.E. (2003). Ribosomal protection proteins and their mechanism of tetracycline resistance. Antimicrob. Agents Chemother..

[50] Barbosa T.M., Scott K.P., Flint H.J. (1999). Evidence for recent intergeneric transfer of a new tetracycline resistance gene, *tet*(W), isolated from *Butyrivibrio fibrisolvens*, and the occurrence of *tet*(O) in ruminal bacteria. Environ. Microbiol..

[51] Kazimierczak K.A., Flint H.J., Scott K.P. (2006). Comparative analysis of sequences flanking tet(W) resistance genes in multiple species of gut bacteria. Antimicrob. Agents Chemother..

[52] Brown K., Zaytsoff S.J., Uwiera R.R., Inglis G.D. (2016). Antimicrobial growth promoters modulate host responses in mice with a defined intestinal microbiota. Sci. Rep..

[53] Public Health Agency of Canada Responsible Use of Medically Important Antimicrobials in Animals. https://www.canada.ca/en/public-health/services/antibiotic-antimicrobial-resistance/animals/actions/responsible-use-antimicrobials.html.

[54] Mirzaagha P., Louie M., Sharma R., Yanke L.J., Topp E., McAllister T.A. (2011). Distribution and characterization of ampicillin- and tetracycline-resistant *Escherichia coli* from feedlot cattle fed subtherapeutic antimicrobials. BMC Mcrobiol..

[55] Gibreel A., Tracz D.M., Nonaka L., Ngo T.M., Connell S.R., Taylor D.E. (2004). Incidence of antibiotic resistance in *Campylobacter jejuni* isolated in Alberta, Canada, from 1999 to 2002, with special reference to *tet*(O)-mediated tetracycline resistance. Antimicrob. Agents Chemother..

[56] Larkin C., Van Donkersgoed C., Mahdi A., Johnson P., McNab B., Odumeru J. (2006). Antibiotic resistance of *Campylobacter jejuni* and *Campylobacter coli* isolated from hog, beef, and chicken carcass samples from provincially inspected abattoirs in Ontario. J. Food Prot..

[57] Rahimi E., Ameri M., Alimoradi M., Chakeri A., Bahrami A.R. (2013). Prevalence and antimicrobial resistance of *Campylobacter jejuni* and *Campylobacter coli* isolated from raw camel, beef, and water buffalo meat in Iran. Comp. Clin. Pathol..

[58] Premarathne J., Anuar A.S., Thung T.Y., Satharasinghe D.A., Jambari N.N., Abdul-Mutalib N.A., Huat J.T.Y., Basri D.F., Rukayadi Y., Nakaguchi Y. (2017). Prevalence and antibiotic resistance against tetracycline in *Campylobacter jejuni* and *C. coli* in cattle and beef meat from Selangor, Malaysia. Front. Microbiol..

[59] Thibodeau A., Fravalo P., Taboada E.N., Laurent-Lewandowski S., Guevremont E., Quessy S., Letellier A. (2015). Extensive characterization of *Campylobacter jejuni* chicken isolates to uncover genes involved in the ability to compete for gut colonization. BMC Microbiol..

[60] De Haan C.P., Kivisto R.I., Hakkinen M., Corander J., Hanninen M.L. (2010). Multilocus sequence types of Finnish bovine *Campylobacter jejuni* isolates and their attribution to human infections. BMC Microbiol..

[61] Backert S., Tegtmeyer N., Cróinín T.O., Boehm M., Heimesaat M.M. (2017). Human campylobacteriosis. Campylobacter: Features, Detection, and Prevention of Foodborne Disease.

[62] Bosilevac J.M., Guerini M.N., Brichta-Harhay D.M., Arthur T.M., Koohmaraie M. (2007). Microbiological characterization of imported and domestic boneless beef trim used for ground beef. J. Food Prot..

[63] Phillips D., Jordan D., Morris S., Jenson I., Sumner J. (2008). A national survey of the microbiological quality of retail raw meats in Australia. J. Food Prot..

[64] Wong T.L., Hollis L., Cornelius A., Nicol C., Cook R., Hudson J.A. (2007). Prevalence, numbers, and subtypes of *Campylobacter jejuni* and *Campylobacter coli* in uncooked retail meat samples. J. Food Prot..

[65] Zhao C., Ge B., De Villena J., Sudler R., Yeh E., Zhao S., White D.G., Wagner D., Meng J. (2001). Prevalence of *Campylobacter* spp., *Escherichia coli*, and *Salmonella* serovars in retail chicken, turkey, pork, and beef from the Greater Washington, D.C., area. Appl. Environ. Microbiol..

[66] Hong J., Kim J.M., Jung W.K., Kim S.H., Bae W., Koo H.C., Gil J., Kim M., Ser J., Park Y.H. (2007). Prevalence and antibiotic resistance of *Campylobacter* spp. isolated from chicken meat, pork, and beef in Korea, from 2001 to 2006. J. Food Prot..

[67] Silva J., Leite D., Fernandes M., Mena C., Gibbs P.A., Teixeira P. (2011). *Campylobacter* spp. as a foodborne pathogen: A review. Front. Microbiol..

