# Erratum: Inglis, G.D., et al. Tetracycline Resistant *Campylobacter jejuni* Subtypes Emanating from Beef Cattle Administered Non-Therapeutic Chlortetracycline Are Longitudinally Transmitted within the Production Continuum but Are Not Detected in Ground Beef. *Microorganisms* 2020, *8*, 23

**DOI:** 10.3390/microorganisms8020273

**Published:** 2020-02-18

**Authors:** G. Douglas Inglis, Jenny F. Gusse, Kathaleen E. House, Tara G. Shelton, Eduardo N. Taboada

**Affiliations:** 1 Lethbridge Research and Development Centre, Agriculture and Agri-Food Canada, 5403-1st Avenue South, Lethbridge, AB T1J 4B1, Canada; Jenny.Gusse@canada.ca (J.F.G.); Kathaleen.House@canada.ca (K.E.H.); Tara.Shelton@canada.ca (T.G.S.); 2 National Microbiology Laboratory, Public Health Agency of Canada, 1015 Arlington Street, Winnipeg, MB R3E 3M4, Canada; Eduardo.Taboada@canada.ca

The authors wish to make the following correction to this paper [1].

On page 21, the copyright should be changed from

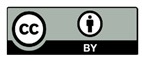
© 2019 by the authors. Licensee MDPI, Basel, Switzerland. This article is an open access article distributed under the terms and conditions of the Creative Commons Attribution (CC BY) license (http://creativecommons.org/licenses/by/4.0/).to the following correct version:

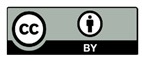
© 2019 by the “Her Majesty the Queen in Right of Canada” for possible open access publication under the terms and conditions of the Creative Commons Attribution (CC BY) license (http://creativecommons.org/licenses/by/4.0/).

The authors would like to apologize for any inconvenience caused to the readers by these changes.
